# Single-Nucleotide Polymorphism Variations Associated With Specific Genes Putatively Identified Enhanced Genetic Predisposition for 305-Day Milk Yield in the Girolando Crossbreed

**DOI:** 10.3389/fgene.2020.573344

**Published:** 2021-01-15

**Authors:** Alex Silva da Cruz, Danilo Conrado Silva, Lysa Bernardes Minasi, Larissa Kamídia de Farias Teixeira, Flávia Melo Rodrigues, Claudio Carlos da Silva, Adriana Santana do Carmo, Marcos Vinicius Gualberto Barbosa da Silva, Yuri Tani Utsunomiya, José Fernando Garcia, Aparecido Divino da Cruz

**Affiliations:** ^1^Mestrado em Genética, Núcleo de Pesquisas Replicon, Escola de Ciências Agrárias e Biológicas, Pontifícia Universidade Católica de Goiás, Goiânia, Brazil; ^2^Curso de Graduação em Medicina Veterinária, Instituto Acadêmico de Ciências Agrárias e Sustentabilidade, Universidade Estadual de Goiás, São Luís de Montes Belos, Brazil; ^3^Escola de Veterinária e Zootecnia, Departamento de Zootecnia, Universidade Federal de Goiás, Goiânia, Brazil; ^4^Empresa Brazileira de Pesquisa Agropecuária, Centro Nacional de Pesquisa de Gado de Leite, Juiz de Fora, Brazil; ^5^Departamento de Apoio a Produção e Saúde Animal, Faculdade de Medicina Veterinária, Universidade Estadual Paulista Júlio de Mesquita Filho, Araçatuba, Brazil

**Keywords:** SNP, dairy cattle, indicine, GWAS, selection

## Abstract

Milk production phenotypes are the main focus of genetic selection in dairy herds, and although there are many genes identified as related to the biology of these traits in pure breeds, little is known about crossbreed animals. This study aimed to identify potential genes associated with the 305-day milk yield in 337 crossbreed Gir × Holstein (Girolando) animals. Milk production records were genotyped for 45,613 single-nucleotide polymorphisms (SNPs). This dataset was used for a genome-wide association study (GWAS) using the 305-day milk yield adjusted for the fixed effects of herd and year and linear and quadratic effects of age at calving (in days) and calving factor averaged per animal. Genes within the significant SNPs were retrieved from the *Bos taurus* ARS-UCD1.2 assembly (bosTau9) for gene ontology analysis. In summary, the GWAS identified 52 SNPs associated [*p* ≤ 10^–4^, false discovery rate (FDR) = 8.77%] with milk production, including NUB1 and SLC24A2, which were previously described as related to milk production traits in cattle. The results suggest that SNPs associated mainly with NUB1 and SLC24A2 could be useful to understand milk production in Girolando and used as predictive markers for selecting genetic predisposition for milk yield in Girolando.

## Introduction

In tropical ecozones, the use of crossbred animals or synthetic breeds has become a viable alternative to overcome the numerous challenges of pasture-based dairy production systems. Girolando, a cross between Gir (*Bos indicus*) and Holstein (*Bos taurus*), has been selected as a suitable breed for dairy farms due to its high adaptability, milk production, and reproductive efficiency in tropical systems (Reis Filho et al., 2015).

Currently, most of the Brazilian dairy herds consist of Girolando cows with their various genetic groups defined based on pedigree-derived ancestry estimates, especially synthetic groups of 5/8 Holstein/Gir, and the breed accounts for over 80% of the milk production in the country (Canaza-Cayo et al., 2016). The heterosis (hybrid vigor) was important for the formation of Girolando and allowed the qualities present in the two breeds to be fixed in the cross.

Dairy fitness, which in turn results in higher milk production per animal, is a complex quantitative trait varying on a continuous scale. It is a worldwide concept that the large range of variation for quantitative traits is due to the number of genes involved in its expression. Thus, the occurrence of various genotypes within a population would reflect variable phenotypic expression subject to a significant effect of the environment on any individual production (Canaza-Cayo et al., 2018).

In this context, it is possible to carry out genome-wide association studies (GWAS) to identify polymorphic chromosomal regions involved in complex traits (Jiang et al., 2019). GWAS allows the identification of DNA markers that strongly correlate to specific traits. DNA markers could be located within gene sequences, revealing candidates potentially involved with the expression of the phenotype of interest. The discovery of markers related to milk production can ensure the accuracy of breeding values and increase the understanding of the genetic control of important traits in economically desired phenotypes (Yin and König, 2019).

Despite the large number of genes previously described as related to the complex trait of milk production in pure breeds, such as Holstein and Jersey cows, very little has been reported in crossbreeds (Canaza-Cayo et al., 2014). Still, the identification of single-nucleotide polymorphism (SNP) affecting milk yield in tropical cattle is of paramount importance to accelerate the rate of genetic change in the dairy industry in developing countries (Daltro et al., 2019).

Therefore, the current study aimed to identify SNP markers associated with the 305-day milk yield in Girolando bred and raised tropically in Brazilian dairy farms.

## Materials and Methods

### Ethics Statement

The study was conducted with data available with the Empresa Brasileira de Pesquisa Agropecuária–Gado de Leite (EMBRAPA Dairy Cattle), and thus, it is exempt from local ethical committee reviews. The Brazilian Association of Girolando Breeders collected phenotypic data and biological samples for animal genetic evaluation, and their members owned the participating herds and voluntarily consented to have their animals included in the study following best practices of veterinary care.

### Single-Nucleotide Polymorphism Genotyping and Quality Control

A total of 337 Girolando cows were genotyped with the Illumina^®^ BovineSNP50 v2 Genotyping BeadChip assay according to the manufacturer’s protocol. Individuals that presented a call rate lower than 90% or that shared identically-by-state over 95% of their alleles with another sample were excluded. Only autosomal SNPs with unique genomic coordinates were analyzed, and markers were removed from the dataset if they did not present a minimum call rate of 90%, minor allele frequency of at least 2%, and Fisher’s exact test *p* value for Hardy–Weinberg equilibrium greater than 1 × 10^–20^. These procedures were performed using customized scripts in *R* v.3.1.1 (available at^1^) and the *GenABEL* v1.8 package (Aulchenko et al., 2007).

### Phenotypic Data Collection and Milk Yield Adjustments

Records of 305-day milk yield (in kg) were available for the genotyped animals, comprising 832 observations. All records were pre-filtered for a minimum lactation period of 30 days and physiological interruption of lactation. First-lactation cows and records of interrupted lactations due to non-physiological reasons (death, loss of the quarter) were excluded from the data. The phenotypic data were adjusted for the fixed effects of herd and year and linear and quadratic effects of age at calving (in days) and days in milk. The total lactation (kg) was adjusted to 305 days by the following formula: (lactation length/305) × milk yield (Associação Brasileira de Criadores de Bovinos da Raça Holandesa [ABCBRH], 1986). The response variable used for associations was composed of the mean of all 305-day milk yield per cow adjusted for the aforementioned environmental effects. The average number of lactations observed per animal was 2.5 ± 1.5, with an interval from 2 to 9 lactations/animal, age at first calving (days) of 1,874.0 ± 758.7, and lactation length (days) of 254.3 ± 70.6.

### Genome-Wide Association Analysis

In order to map putative quantitative trait loci (QTLs), associations between markers and phenotypes were tested using the following single-marker linear regression model:

$$y=1_{n}\,\mu +x\,b+e$$

where *y* is the column vector of phenotypes, 1_*n*_ is a vector of 1 s, μ is the overall mean, *b* is a column vector of unobserved allele substitution effects, *x* is a vector of genotypes (coded as 0, 1, or 2 Illumina B alleles) relating observations in *y* to vector *b*, and *e* is the column vector of random residual effects, assumed $e∼N\,\left(0,\sigma_{e}^{2}\right)$. This model was fitted using the ordinary least squares method. Markers were prioritized for functional annotation conditional on suggestive or significant values of *p* < 10^–4^ and *p* < 10^–5^, respectively. The expected false discovery rate (FDR) was computed as:

F⁢D⁢R=k⁢α/s

where *k* is the total number of markers being tested, α is the significance level adopted, and *s* is the number of markers declared significant at α. These procedures were performed using customized scripts in *R* v.3.1.1 [available at (see text footnote 1)] and the *GenABEL* v1.8 package [1].

### Partitioning of Marked Variance

Ideally, the variance explained by causal quantitative trait nucleotides (QTNs) can be estimated *via* the linear model:

$$y=1_{n}\,\mu +g+e$$

where $g=\sum_{i=1}^{k}x_{i}\,b_{i}$is the vector of random breeding values. These values represent the sum of the effects of all inherited QTNs and are assumed $g∼N\,\left(0,K\,\sigma_{g}^{2}\right)$, where *K* is the kernel matrix of additive genetic relationships between pairs of individuals at QTNs, and $\sigma_{g}^{2}$ is the variance component attributed to the causal variants (i.e., additive genetic variance). An alternative parameterization of this model under the Reproducing Kernel Hilbert Spaces (RKHS) framework considers the change of variable *g=Kc*, which results in $c∼N\,\left(0,K^{-1}\,\sigma_{c}^{2}\right)$, where variance components $\sigma_{g}^{2}$ and $\sigma_{c}^{2}$ are interchangeable.

As QTNs are not known in practice, genome-wide markers are often used as proxies to indirectly capture the effects of causal loci, and matrix *K* is computed from high-density SNP data. Different relationship matrices based on specific SNP sets, such as markers clustered by chromosomes, can also be computed to partition the additive genetic variance onto variance due to groups of markers [3]. Here, in order to estimate the variance due to putative major QTLs and remaining polygenic effects, we partitioned the additive genetic variance between genome-wide significant and non-significant markers by fitting, as described by Colli et al. (2018).

$$y=1_{n}\,\mu +K_{Q\,T\,L}\,c_{Q\,T\,L}+K_{G}\,c_{G}+e$$

where subscripts QTL and G represent significant and non-significant genome-wide SNPs. Model parameters were estimated using the Gibbs sampling algorithm implemented in the *BGLR* v1.0.3 package in R [4]. Normal priors were assigned to random effects, and a flat prior was assigned to the overall mean. Variance components were assumed *a priori* scaled inverse chi-square distributed with ν = 5 degrees of freedom and scale parameter *S* = *var*(*y*)(1−*R*^2^)(ν + 2), where *R*^2^ is the proportion of phenotypic variance *a priori* assigned to the random effects. We assumed *R*^2^ = 0.5 for the residual variance, and the unexplained variance was equally assigned to the remaining variance components. A single Markov chain with a length of 100,000 iterations was used. The burn-in period was set at 10,000 iterations and the thinning interval at 100 iterations.

For Gene Ontology analysis, genes within significant SNPs were considered; if the SNP was located in the intergenic region (i.e., not assigned to any gene), we selected the closest gene from the marker according to the ARS-UCD1.2 reference genome (Lehne et al., 2011; Buzanskas et al., 2017; Martínez et al., 2017; Cai et al., 2019). Gene names and coordinates were retrieved from the Ensembl Genes 101 database using the BioMart tool. SNP probe coordinates were migrated from UMD 3.1 (bosTau6) to ARS-UCD1.2 (bosTau9) assembly using UCSC liftOver^2^.

## Results

### Genotype Quality Control and Phenotype Adjustment

From an initial set of 404 cows, 54,609 SNPs were collected. However, at best, 337 animals and 45,622 markers passed all filtering criteria. The average (± SD) number of parities observed per animal, age at calving (in days), and days in milk were 2.47 ± 1.47, 1,874.0 ± 758.72, and 254.3 ± 70.6, respectively. The averages (± SD) for minimum and maximum milk yields were 4,563.0 ± 2,280.83 kg and 316.0 ± 13.88 kg, respectively.

### Genome-Wide Association

**FIGURE 1:**
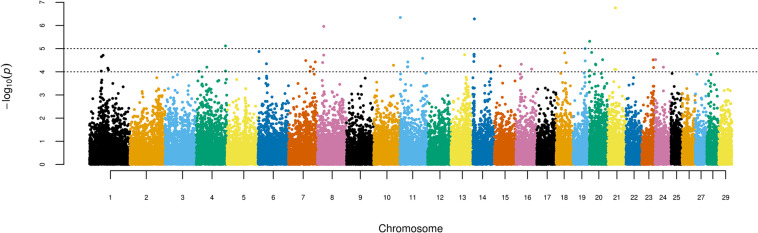
Manhattan plot displaying the results of the genome-wide analysis of milk production adjusted to 305 days in Girolando cattle.

Figure 1 shows the results of the single-marker linear regression analysis (*p* = 10^–4^ and FDR 8.77%), indicating 52 significant SNPs. When the threshold was set to *p* = 10^–5^, seven significant markers remained (FDR 6.52%). Significant associations were detected in 18 out of the 29 bovine autosomes. Thirty-five of the 52 SNPs identified *via* the GWAS in Girolando cows are associated with previously described genes of the bovine genome (FDR 8.77% and *p* = 10^–4^; Table 1), while seven of these SNPs (*p* = 10^–5^) are associated with four genes in the physical map of the bovine genome, namely, Solute Carrier Family 24 Member 2 (SLC24A2 and BTA8), Negative Regulator of Ubiquitin-Like Protein 1 (NUB1 and BTA4), KH RNA Binding Domain Containing, Signal Transduction Associated 3 (KHDRBS3 and BTA14), and Membrane-Associated Ring-CH-Type Finger 10 (MARCHF10 and BTA19).

**TABLE 1:** Single-nucleotide polymorphisms (SNPs) distributed in genes in the physical map of the bovine genome identified by genome-wide association study (GWAS) for 305-day milk yield using a 50 K SNP chip in Girolando cattle from Brazilian herds.

SNP			Gene				
Marker	Chrom^1^	Position	Pos^2^	End Pos^3^	Name	Distance	Threshold
BTB-00213370	4	113,889,552	113,890,451	113,925,971	NUB1	899	≤10^–5^
ARS-BFGL-NGS-71395	8	24,741,844	24,532,716	24,812,024	SLC24A2	*	
ARS-BFGL-BAC-12761	14	6,526,644	6,400,724	6,544,685	KHDRBS3	*	
ARS-BFGL-NGS-414	19	47,280,939	47,110,902	47,203,442	MARCHF10	77,498	
ARS-BFGL-NGS-78259	1	52,620,931	52,712,128	52,777,176	CD47	91,197	10^–4^
ARS-BFGL-NGS-97095	1	70,823,693	70,809,680	70,827,658	SLC51A	*	
ARS-BFGL-NGS-114968	1	73,398,276	73,362,759	73,365,438	HES1	32,839	
Hapmap41216-BTA-27440	4	11,414,051	11,330,484	11,345,143	BET1	68,909	
ARS-BFGL-NGS-100194	4	113,945,281	113,931,167	113,956,890	WDR86	*	
BTB-01790614	6	3,741,604	3,557,585	3,590,521	ANXA5	151,084	
Hapmap27294-BTC-032117	6	31,425,251	30,780,273	31,540,156	GRID2	*	
ARS-BFGL-NGS-42452	7	65,274,641	65,295,989	65,330,574	FAM114A2	21,348	
BTB-00960162	7	81,611,128	81,401,606	81,711,503	SSBP2	*	
BTB-01363189	7	83,818,631	83,846,108	84,324,020	EDIL3	27,477	
Hapmap47490-BTA-108189	7	95,156,038	95,160,550	95,160,660	U6	4,512	
Hapmap48479-BTA-80447	7	101,857,168	101,896,775	102,084,454	PAM	39,607	
Hapmap53914-rs29021936	8	20,554,560	20,303,380	20,443,479	ELAVL2	111,082	
BTB-01052867	8	24,769,391	24,532,716	24,812,024	SLC24A2	*	
ARS-BFGL-NGS-101534	11	28,896,192	28,928,747	28,933,551	TMEM247	32,555	
Hapmap38097-BTA-117206	11	30,218,982	30,143,409	30,256,247	FBXO11	*	
BTB-01468914	11	30,305,738	30,143,409	30,256,247	FBXO11	49,492	
ARS-BFGL-BAC-16207	11	88,018,340	88,013,833	88,030,303	ITGB1BP1	*	
ARS-BFGL-NGS-78318	14	4,301,610	4,273,909	4,274,015	U6	27,596	
ARS-BFGL-NGS-114178	14	4,606,723	4,388,017	4,643,810	FAM135B	*	
ARS-BFGL-NGS-29032	16	59,897,334	59,915,002	59,925,320	TEX35	17,668	
ARS-BFGL-NGS-76555	18	33,930,881	33,880,439	33,880,545	U6	50,337	
ARS-BFGL-NGS-55014	18	40,088,744	40,063,356	40,063,445	5S_rRNA	25,300	
ARS-BFGL-BAC-35051	19	47,561,236	47,441,495	47,758,825	TANC2	*	
ARS-BFGL-NGS-101925	20	1,314,256	447,373	1,163,533	SLIT3	150,724	
ARS-BFGL-NGS-41186	20	9,491,210	9,401,263	9,494,370	MAP1B	0	
ARS-BFGL-NGS-30073	20	24,676,265	24,638,190	24,638,869	HSPB3	37,397	
ARS-BFGL-NGS-110286	20	25,681,663	25,580,150	25,586,931	FST	94,733	
BTB-01132138	20	52,125,535	51,049,217	51,567,420	CDH12	558,116	
ARS-BFGL-NGS-110044	21	30,829,086	30,828,476	30,879,177	IREB2	*	
ARS-BFGL-NGS-104353	23	45,452,240	45,452,427	45,553,179	GCNT2	187	
ARS-BFGL-BAC-36400	23	47,188,127	47,158,325	47,179,853	SLC35B3	8,275	
ARS-BFGL-NGS-30248	24	2,317,459	2,224,463	2,328,579	MBP	*	
ARS-BFGL-NGS-100144	24	32,714,013	32,711,020	32,946,065	LAMA3	*	
UA-IFASA-6255	28	41,533,590	41,486,119	41,544,234	BMPR1A	*	

^1^Bos taurus autosome; ^2^gene initial position (bp); ^3^gene end position (bp); and *intragenic SNPs.

Table 2 presents the variance component estimates of the models, partitioning the additive genetic variance between markers declared significant at different levels (10^–4^ and 10^–5^) and the remaining markers. The top-scoring seven SNPs explained 14.4% of the adjusted phenotypes alone, whereas all 52 top-scoring SNPs together explained 28.7% of the variance in the adjusted phenotypes. Altogether, genome-wide SNPs could explain as much as 49% of the phenotypic variance.

**TABLE 2:** Significant (p < 10^–4^) single-nucleotide polymorphisms (SNPs) identified by the genome-wide association study of 305-day milk yield in Girolando cattle.

SNP	BTA^1^	Position	N^2^	MAJ^3^	p value
BTB-01462011	1	45,581,665	337	A	9.35e-05
ARS-BFGL-NGS-114968	1	73,398,276	332	B	8.19e-05
ARS-BFGL-NGS-97095	1	70,823,693	337	B	6.98e-05
ARS-BFGL-NGS-67684	1	46,267,521	337	B	2.23e-05
ARS-BFGL-NGS-78259	1	52,620,931	337	A	1.94e-05
Hapmap41216-BTA-27440	4	11,414,051	337	A	9.62e-05
ARS-BFGL-NGS-100194	4	113,945,281	335	A	9.29e-05
BTA-70284-no-rs	4	42,282,130	336	A	6.30e-05
BTB-00213370	4	113,889,552	333	B	7.63e-06
Hapmap27294-BTC-032117	6	31,425,251	336	B	4.50e-05
BTB-01790614	6	3,741,604	337	B	1.33e-05
BTB-00960162	7	81,611,128	337	B	9.50e-05
Hapmap47490-BTA-108189	7	95,156,038	337	B	7.62e-05
BTB-01363189	7	83,818,631	337	A	6.12e-05
Hapmap48479-BTA-80447	7	101,857,168	337	B	3.76e-05
ARS-BFGL-NGS-42452	7	65,274,641	337	B	3.26e-05
Hapmap53914-rs29021936	8	20,554,560	337	B	3.97e-05
BTB-01052867	8	24,769,391	337	A	1.90e-05
ARS-BFGL-NGS-71395	8	24,741,844	337	B	1.09e-06
ARS-BFGL-NGS-111205	10	78,005,122	337	A	5.20e-05
ARS-BFGL-NGS-101534	11	28,896,192	337	B	6.09e-05
BTB-01468914	11	30,305,738	337	B	6.04e-05
Hapmap38097-BTA-117206	11	30,218,982	337	B	3.75e-05
ARS-BFGL-BAC-16207	11	88,018,340	337	B	2.62e-05
ARS-BFGL-NGS-62942	11	897,007	337	B	4.52e-07
ARS-BFGL-NGS-14448	13	53,296,446	337	A	1.83e-05
ARS-BFGL-NGS-78318	14	4,301,610	337	B	3.59e-05
Hapmap51078-BTA-87682	14	5,668,065	337	B	2.14e-05
ARS-BFGL-NGS-114178	14	4,606,723	337	A	1.80e-05
ARS-BFGL-BAC-1212	14	5,614,291	337	A	1.75e-05
ARS-BFGL-BAC-12761	14	6,526,644	337	B	5.20e-07
ARS-BFGL-NGS-102765	15	23,091,526	337	B	5.58e-05
ARS-BFGL-NGS-29032	16	59,897,334	337	B	7.62e-05
BTB-01422500	16	20,297,366	336	B	4.72e-05
ARS-BFGL-NGS-55014	18	40,088,744	336	B	4.02e-05
ARS-BFGL-NGS-76555	18	33,930,881	337	B	1.51e-05
ARS-BFGL-BAC-35051	19	47,561,236	337	B	3.37e-05
ARS-BFGL-NGS-414	19	47,280,939	337	B	9.95e-06
ARS-BFGL-NGS-101925	20	1,314,256	337	A	8.79e-05
ARS-BFGL-NGS-110286	20	25,681,663	337	A	4.98e-05
ARS-BFGL-NGS-30073	20	24,676,265	337	B	4.67e-05
BTB-01132138	20	52,125,535	337	A	3.00e-05
ARS-BFGL-NGS-41186	20	9,491,210	337	B	1.45e-05
ARS-BFGL-NGS-20999	20	1,708,332	337	A	4.82e-06
ARS-BFGL-NGS-110044	21	30,829,086	335	A	7.97e-05
ARS-BFGL-NGS-119292	21	27,584,961	337	A	7.86e-05
Hapmap38535-BTA-85920	21	29,955,227	335	A	1.73e-07
ARS-BFGL-BAC-36400	23	47,188,127	337	B	6.53e-05
ARS-BFGL-NGS-104353	23	45,452,240	334	B	3.03e-05
ARS-BFGL-NGS-100144	24	32,714,013	337	B	6.31e-05
ARS-BFGL-NGS-30248	24	2,317,459	337	B	2.98e-05
UA-IFASA-6255	28	41,865,345	337	B	1.63e-05

*^1^Bos taurus autosome; ^2^Number of animals; and ^3^Major allele frequency.*

## Discussion

Considering that bovine parity is a quantitative trait, it is very likely that the genes identified in association with 305-day milk yield in Girolando account for a small fraction of the total genetic variance for milk yield. Nevertheless, the findings reported here are essential in the context of the biology and functional characterization of milk production in Girolando, the most common industrial dairy crossbreed in Brazil.

The *SLC24A2* gene encodes member 2 of the solute carrier family 24 of transporter proteins. The SLC24A family comprises six mammalian protein subtypes that exchange cellular calcium and potassium with extracellular sodium (Altimimi et al., 2010). In humans, multiple variants of SLC24A2 have been described. The protein is considered to have a neuroprotective role and is found primarily in the retina and brain (Cuomo et al., 2007), additionally, in cattle, the process of sodium:calcium exchange in the photoreceptors was described in 1986 (Schnetkamp, 1986). However, there is no further information regarding the genetic role of SLC24A2 in milk production. Recent studies have demonstrated that the genes of the SLC24A family extrude calcium and potassium ions out of the cell by the entry of sodium ions (Altimimi et al., 2010). This process was also demonstrated in the uterine endometrium during the estrous cycle and pregnancy in pigs (Choi et al., 2014). In our study, two SNPs were linked to the *SLC24A2* gene, which was significantly associated with milk production in the Girolando crossbreed (Table 1).

In humans and bovines, the *NUB1* gene encodes a crucial ubiquitin-like protein that operates in cell cycle progression for tissue maintenance (Akey et al., 2002). The *NEDD8* plays a vital role in cell cycle control and embryogenesis, and the *AIPL1* is a chaperone controlling nuclear transport activity (Arcot et al., 2015; Lucariello et al., 2013). The gene *NUB1* interacts primarily with multiple potential ubiquitin genes, and it has been demonstrated that *NEDD8* is expressed in bovine milk somatic cells and that its expression increases gradually throughout the lactation cycle, doubling its expression from early to late lactation in Holstein cows (Wickramasinghe et al., 2012). Thus, *NUB1* may play an essential role in controlling milk yield in cattle.

One relevant observation is that both *NUB1* and *SLC24A2* are primarily expressed within the photoreceptors of the retina (Akey et al., 2002). The interactions established by the proteins encoded by these two genes are essential for capturing light through the retina, and their role is dependent on their level of expression (Kirschman et al., 2010). According to Dawkins (2006), in the tropics, the light intensity remains relatively constant throughout the year but varies in temperate climates according to seasonal changes. In cattle, the response to light stimuli occurs through the hypothalamus–pituitary axis, and the eyes are the primary recipients of brightness (Valenzuela-Jiménez et al., 2015). Several authors (Yaegashi et al., 2012; Tsang et al., 2014) suggest that the manipulation of the photoperiod could be a useful variable for increasing milk production in cattle as it affects prolactin secretion in mammals. Valenzuela-Jiménez et al. (2015) found an increase of approximately 15% in the lactation of Holstein cows raised in a controlled environment with average daylight of 16 h when compared to an environment with a shorter photoperiod of 8 h. Likewise, Dahl et al. (2012) found a higher growth rate and increased milk production in the first lactation of heifers exposed to longer photoperiods.

The aforementioned reports support the role of *NUB1* as a potential functional candidate gene for increased milk production in cattle, both by acting as a modulator of brightness perception in the retina and by controlling gene expression owing to ubiquitination. The SLC24A2 gene, also expressed in the retina, may be involved in light capture mechanisms, thereby influencing milk yield. Both mechanisms have been well-investigated in Holstein cows, a breed that contributes a substantial part of the Girolando’s genomes. Thus, artificial selection may have indirectly contributed to the role of *NUB1* and *SLC24A2* in modulating milk production and yield in Brazilian climates, characterized by longer photoperiods throughout the year.

The *MARCHF10* gene is a member of the MARCH family of membrane-bound E3 ubiquitin ligases to target lysines in substrate proteins and participates in signaling vesicular transport between membrane compartments (Morokuma et al., 2007). The *KHDRBS3* has a role in blood–tumor barrier (BTB) that severely restricts the efficient delivery of antitumor drugs to cranial glioma tissues (Wu et al., 2019). The functional effects of *MARCHF10* and *KHDRBS3* have been described in humans, with no evidence of functions for cattle lactogenesis.

The 52 SNPs identified in the current GWAS with an FDR of 8.77% for the threshold at 10^–4^ were linked to several genes that were not previously associated with milk production, and their cellular roles vary from DNA binding to transcription factors that regulate cell growth and proliferation (Poitras et al., 2008; Huang et al., 2011; Aslibekyan et al., 2012; Xiao et al., 2012; Lemos et al., 2016; Grigoletto et al., 2020), and an investigation of the effect of the beef fatty acid profile detected in Angus in the 778 K analysis revealed at least two biologically relevant genes, namely, *KHDRBS* and FAM135 (Table 1), previously associated with livestock feed efficiency, residual average daily gain, and adipogenesis (Seabury et al., 2017). The putative functional role of these genes in milk production, if any, remains to be identified. If identified, the GWAS results indicate the potential for SNP-based genomic selection for genetic improvement of Girolando crossbred cattle.

Our data suggest that *NUB1* and *SLC24A2* are important genes for understanding milk production in Girolando and lay a preliminary foundation for designing future follow-up studies regarding this trait in the crossbreed. In addition, the identified SNPs could be used as potential markers to putatively identify enhanced genetic predisposition for milk yield in the most common industrial dairy crossbreed in Brazil.

## Data Availability Statement

The data analyzed in this study was obtained from an agreement between Embrapa (Empresa Brasileira de Pesquisa Agropecuária), CRV, and Zoetis. Thus, legal and privacy restrictions prevent data from becoming publicly available. However, requests to access these datasets should be directed to Dr. MS at marcos.vb.silva@embrapa.br.

## Ethics Statement

Ethical review and approval was not required for the animal study because the present study was exempt of the local ethical committee evaluation as genomic DNA was extracted from stored hair of animals from commercial herds. Written informed consent was obtained from the owners for the participation of their animals in this study.

## Author Contributions

ACr, DS, and ACa wrote the manuscript. ApC, CS, MS, and JG conceived the study. YU, ACa, and MS contributed to data production and quality control. FR, DS, and YU performed data analysis. LM and LF contributed to the interpretation of the results. ApC and MS provided samples or funded part of the analyses. All authors read, made corrections, contributed, and approved the manuscript.

## Conflict of Interest

The authors declare that the research was conducted in the absence of any commercial or financial relationships that could be construed as a potential conflict of interest.

## Footnotes

1 http://www.r-project.org/

2 http://hgdownload.cse.ucsc.edu/admin/exe/
